# Scar Endometriosis: A Rare Cause of Abdominal Pain

**DOI:** 10.3390/dermatopathology9020020

**Published:** 2022-05-05

**Authors:** Rohit Nepali, Santosh Upadhyaya Kafle, Tarun Pradhan, Jiba Nath Dhamala

**Affiliations:** 1 Department of Obstetrics and Gynaecology, Birat Medical College and Teaching Hospital, Buddhiganga-02, Morang, Biratnagar 56617, Nepal; rohit.nepali1998@gmail.com (R.N.); tarunsriti@gmail.com (T.P.); 2 Department of Pathology, Birat Medical College and Teaching Hospital, Buddhiganga-02, Morang, Biratnagar 56617, Nepal; pathologysantosh@gmail.com

**Keywords:** scar endometriosis, Pfannenstiel incision, lower segment cesarean section

## Abstract

Scar endometriosis or incisional endometriosis is the presence of endometrial tissues with glands in the previous incision or scar. Its overall estimated incidence after post-cesarean and post-hysterectomy is 0.03–0.4% and 1.08–2%, respectively. The patient presents with non-specific symptoms such as cyclical abdominal pain at the site of a previous surgical incision and scar and an abdominal lump with a cyclical increment in size, which is tender. The diagnosis is made only after the surgical excision with confirmation by histopathological analysis. We present the case of a 31-year-old female complaining of cyclical abdominal pain and a lump on the right side of a Pfannenstiel incision for five months. She had undergone two Lower Segment Caesarean Sections (LSCSs); the last surgery was eight months prior. Surgical excision was planned with the corresponding clinical features and radiological data. After the surgical excision, the sample was sent for histopathological examination, and scar endometriosis was diagnosed.

## 1. Introduction

Endometriosis is defined as functional endometrial glands with stroma outside the uterus, which is estimated to affect almost 10% of the reproductive age groups (15–49 years [1]. It usually occurs around the uterus and uterine ligaments; however, extra pelvic sites, though rare, occur in the lungs, brain, urinary tracts, abdominal wall, spleen, gastrointestinal tracts, and previous surgical scars such as a previous cesarean scar, episiotomy scar, ectopic pregnancies, hysterectomy, salpingostomy, or herniorrhaphy [2,3]. The presence of endometrial glands with stroma above the peritoneum (skin, subcutaneous tissue, muscle, scar) is collectively known as abdominal wall endometriosis [2,4].

Cesarean scar endometriosis is the most common abdominal wall endometriosis, with an estimated incidence of 0.03–0.4% [4,5,6,7,8]. The pathophysiology for its occurrence is iatrogenic implantation (implantation theory), where the refluxed endometrial tissue on obstetrics and gynecological surgical manipulation is implanted on the ectopic sites [2,4]. Under the proper hormonal influence, the endometrial tissue proliferates and leads to cesarean scar endometriosis [2,4,7,8,9,10,11,12]. Additionally, the surrounding primitive pluripotent mesenchymal cells may undergo specialized metaplasia to form cesarean scar endometriosis [7,8].

## 2. Case Presentation

A 31-year-old female Parity 2 Living 1 (P2L1) presented to the Birat Medical College and Teaching Hospital outpatient department with complaints of pain and swelling over the right side of a previous Pfannenstiel incision site. She had no other significant medical history except for two Lower Segment Cesarean Sections (LSCSs) 8 years and eight months prior. The pain was cyclical, occurring 2–3 days before menstruation, peaking during the menstruation, and gradually subsiding within three days after menstruation. She had to take analgesics to control the pain. She noticed swelling over the right side of the previous incision site that was progressive, non-compressible, and non-reducible.

On physical examination, there was a healed Pfannenstiel incision scar. A lump approximately 3 × 2 cm at the right side of the healed scar was nodular, tender, ill-defined, slightly fixed, and non-mobile, with no surrounding skin changes or herniation of abdominal content.

Ultrasonography suggested an ill-defined heterogeneous heteroechoic focal lesion showing the presence of mild internal vascularity in the subcutaneous plane of the lower abdominal wall scar.

Based on history, clinical examination, and ultrasonography, a diagnosis of scar endometriosis was made, and wide local excision was planned.

**Figure 1:**
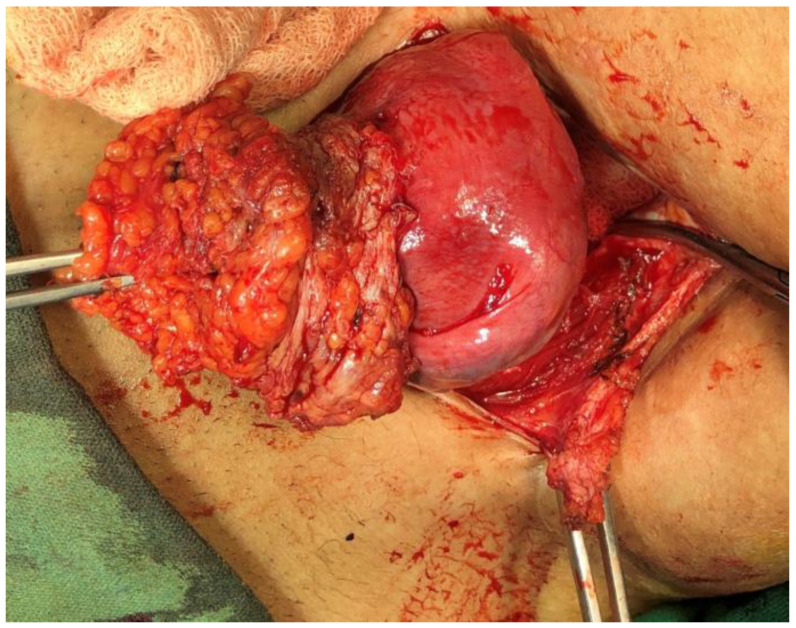
A lump of 5 × 3 cm encased in the fibrosis tissue, extending from the subcuticular plane, infiltrating the rectus sheath, and extending up to the anterior surface of the uterus.

**Figure 2:**
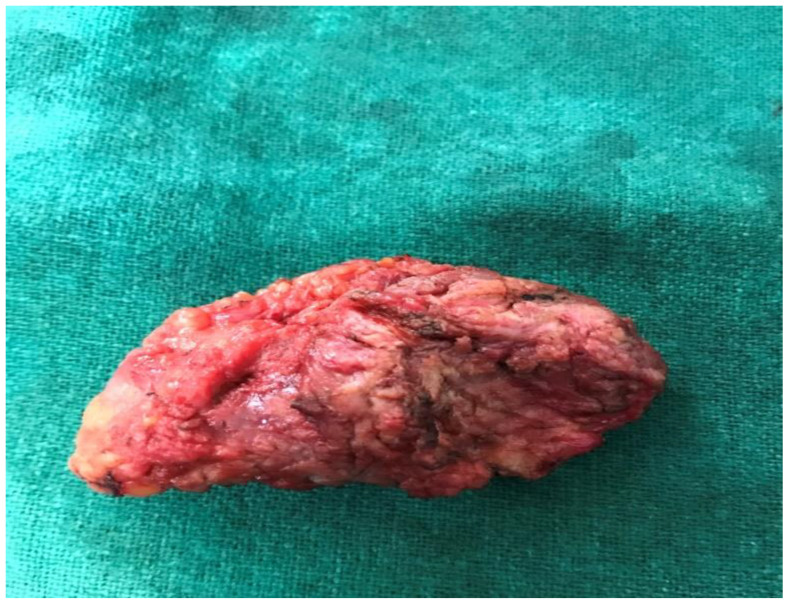
Wide local excision 6 × 4 × 1.4 cm; grayish-brown to black in color.

Intra-operatively, we found a lump 5 × 3 cm encased in fibrosis tissue extending from the subcuticular plane, infiltrating the rectus sheath, and extending up to the anterior surface of the uterus (Figure 1). Wide local excision 6 × 4 × 1.4 cm (Figure 2) was performed, and the tissue was sent for histopathological analysis. Postoperatively, the patient was stable and discharged on the fourth postoperative day.

**Figure 3:**
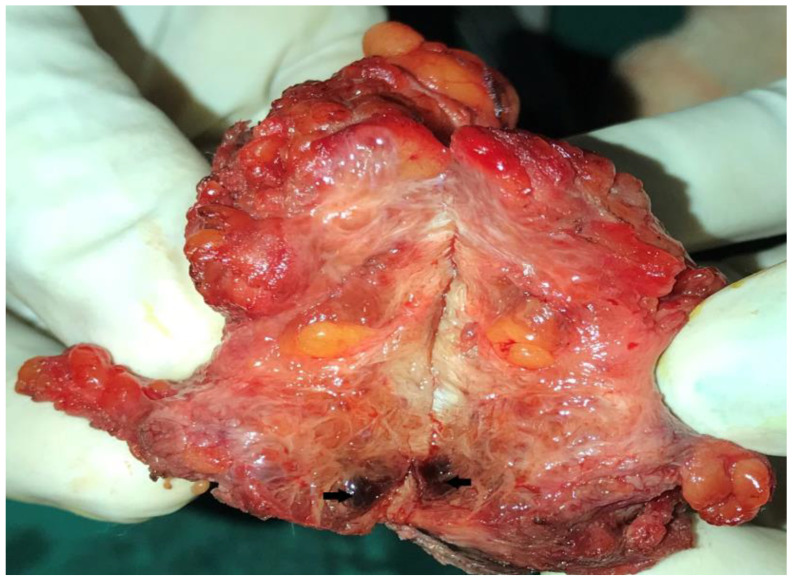
The cut surface was grayish-white to solid brown homogeneous area with focal blackish discoloration.

On gross examination, the outer surface was partially cut open and grayish brown to black (Figure 2). On further cut opening, the cut surface was a grayish-white to solid brown homogeneous area with focal blackish discoloration (Figure 3).

**Figure 4:**
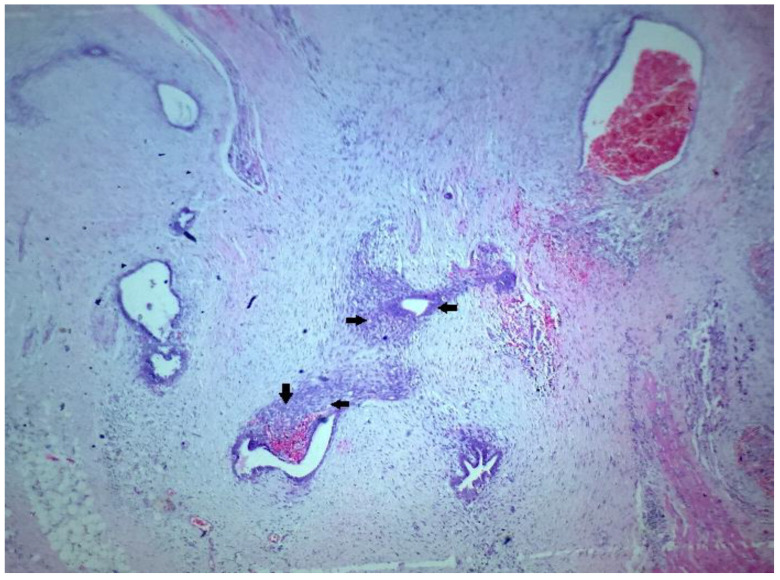
(H&E stain × 40): Scattered numerous endometrial glands along with stroma; a few various calibered, congested, as well as dilated vascular channels; and mixed inflammatory cells predominantly comprising mature lymphocytes, histocytes, and plasma cells embedded against a background of fibro collagenous, muscular, and adipose stroma.

**Figure 5:**
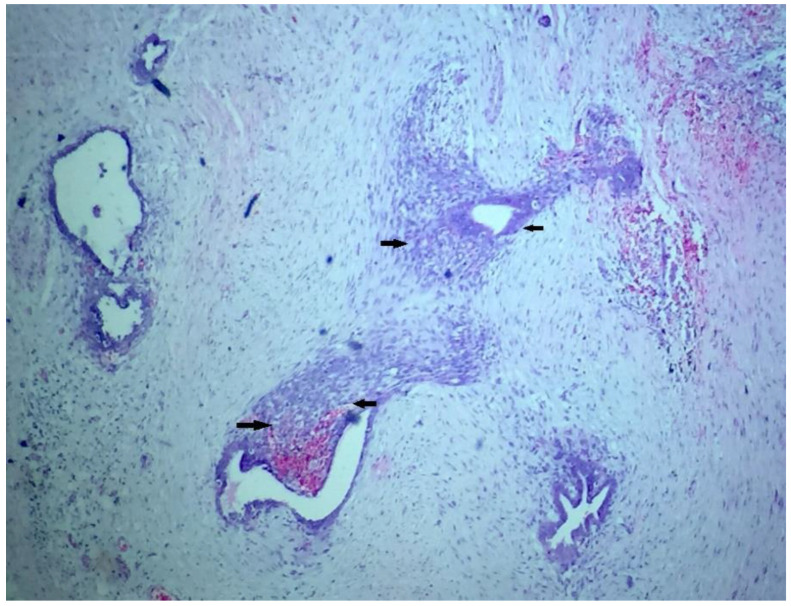
(H&E stain × 60): High-power view showing the scattered endometrial glands and stroma.

**Figure 6:**
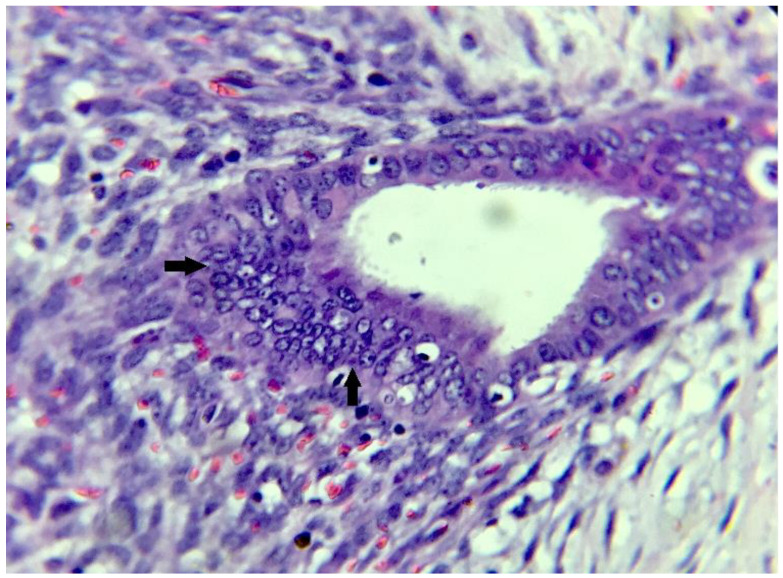
Endometrial gland with stroma.

On microscopic examination, multiple sections showed scattered numerous endometrial glands along with stroma (Figure 4, Figure 5 and Figure 6) a few various calibered, congested, as well as dilated vascular channels; and mixed inflammatory cells predominantly comprising mature lymphocytes, histiocytes, and plasma cells embedded against a background of fibro-collageneous, muscular, and adipose stroma (Figure 4 and Figure 5). The diagnosis of scar endometriosis was made based on a histopathological report.

## 3. Discussion

Endometriosis is defined as the presence of functional endometrial glands with stroma outside the uterus [1]. It usually occurs around the uterus, such as in the ovaries, uterine ligaments, cul-de-sac, and pelvic peritoneum, but can occur in almost all body parts [2,3,4]. Abdominal wall endometriosis is one of the major extra pelvic sites for endometriosis, which occurs after abdominal, obstetric, and gynecological surgeries, among which cesarean section is the most common one [4,7,8].

Nominato et al. analyzed 72 cases, and 66.6% had lesions in the abdominal wall, and 63.8% were found in the cesarean section in the surgical procedures [2].

The widely accepted theory for its occurrence is the iatrogenic implantation theory, where a refluxed endometrial tissue from gynecological and obstetrical surgical procedures is implanted on the incision site, and under proper hormonal influence, endometrial tissue proliferates and forms scar endometriosis [2,4].

Laxmi et al. analyzed the growth rate of LSCS and found a sharply rising trend of LSCS rates from 20 to 81% in different private hospitals in Nepal, suggesting over-medicalization in childbirth and obstetric care [13]. Concerning the rising Caesarean section rate, scar endometriosis may occur more frequently than generally assumed; hence, practitioners’ attention should be on the early diagnosis, treatment, and prevention of scar endometriosis. Although the duration for the occurrence of clinical symptoms varies from 3 months to 15 years, in our case, it occurred 8 months after the second LSCS surgery [6].

The common symptoms include cyclical pain, swelling worsening during the menstrual cycle, and rarely bleeding in the lesion area; however, there may be non-cyclical symptoms. Due to its nature, it is often misdiagnosed as other surgical issues such as an incisional hernia, abscess, suture granuloma, abdominal wall tumor, hematoma, or neuroma [6,14]. So, its diagnosis is entirely based on a high index of suspicion with proper history-taking and clinical examination.

Malignant transformation has also been found, among which endometrioid carcinoma is the most common one [14].

Other modalities, such as Ultrasonography, Computed Tomography (CT), and Magnetic Resonance Imaging (MRI), might help establish a diagnosis and facilitate surgical excision but not diagnostics [3,6,7,14]. Fine-Needle Aspiration Cytology (FNAC) is an essential diagnostic tool that can help in establishing a diagnosis and eliminating the malignancy [14].

Medical therapy such as Gn-RH agonist, Progesterone, oral contraceptive pills, and danazol have been tried, but they only allow partial relief and cause recurrence quickly [3,6,15].

The diagnosis is confirmed by the histopathological examination of the excised tissue. The definite treatment is surgical excision with at least 5–10 mm free margin of surrounding healthy tissue. Care should be taken during the excision so as not to rupture the mass to avoid re-implantation [3,6,10,15]. The diagnosis is confirmed by the histopathological examination of the excised tissue.

## 4. Conclusions

Scar endometriosis is one of the major extra pelvic sites for scar endometriosis, among which a cesarean scar is the most common. Concerning the rising rate of lower segment cesarean section, scar endometriosis can occur more frequently; hence, attention should be paid to the early diagnosis, treatment, and prevention of scar endometriosis.

## Data Availability

Not applicable.
